# Survival of bovine digital dermatitis treponemes on hoof knife blades and the effects of various disinfectants

**DOI:** 10.1136/vr.105406

**Published:** 2019-11-19

**Authors:** Amy Gillespie, Stuart D Carter, Roger W Blowey, Nicholas Evans

**Affiliations:** 1 Infection Biology, Institute of Infection and Global Health, University of Liverpool Leahurst Campus, University of Liverpool, Neston, UK; 2 Wood Veterinary Group, Gloucester, UK

**Keywords:** Dairy cattle, Digital dermatitis, Treponemes, Disinfection, Hoof knives

## Abstract

**Background:**

Bovine digital dermatitis (BDD) is a painful infectious foot disease of cattle, and much evidence implicates a pathogenic role for treponemes. This study measured the survival of BDD treponemes on hoof knife blades and tested the efficacy of relevant disinfectants under laboratory conditions.

**Methods:**

Two strains of BDD treponemes were applied to hoof knife blades under aerobic conditions. Swabs were taken at different time points (10 minutes, one hour, two hours, four hours and 18 hours) and again after 20-second disinfection time with one of five disinfectants. Swabs were used directly for nested PCR to detect treponemes or inoculated for anaerobic growth, and subsequently examined using phase contrast microscopy and PCR.

**Results:**

BDD treponeme DNA was detectable by nested PCR at all survival time points, and these organisms were culturable from hoof knives for two hours after exposure under aerobic conditions in the laboratory. Three of the five disinfectants—1 per cent volume per volume (v/v) FAM30^®^, 2 per cent weight per volume (w/v) Virkon^®^ or 2 per cent (v/v) sodium hypochlorite—were effective at preventing visible growth of treponemes following 20-seconds contact, and 1 per cent (v/v) FAM30^®^ also prevented detection of treponemes by PCR.

**Conclusion:**

Treponeme viability of two hours under aerobic conditions suggests BDD treponemes could be transmitted between cows on hoof knives. It is therefore important to apply a disinfection protocol during foot-trimming; the authors have identified three common disinfectants that may be suitable.

## Introduction

Bovine digital dermatitis (BDD) is an infectious foot disease of cattle, affecting a large proportion of dairy herds worldwide.^1^ Globally, three distinct phylogroups of treponemes have been isolated from BDD lesions,^2 3^ and these have been demonstrated as highly associated with BDD lesions.^4 5^

Previous work has identified that hoof knives used to routinely trim cows’ feet become contaminated with these infectious bacteria.^6^ Precise BDD transmission routes are not fully understood; however, treponeme contamination of blades during trimming may be relevant if organisms survive long enough to be transferred to another foot.

Disinfection of hoof knives between animals is not always carried out, and there is currently no validated practical disinfection regimen. Epidemiological studies (USA) have considered the foot-trimming biosecurity risks for BDD and identified the use of foot-trimmers who trimmed on other farms and lack of hoof-trimming equipment washing between cows as BDD risk factors.^7^ External foot-trimmer use was also implicated in increased BDD prevalence in New Zealand herds.^8^ Both studies advocated disinfection of foot-trimming equipment. A range of disinfectants have been tested *in vitro* against a BDD treponeme isolate, with minimum inhibitory concentrations (MICs) and minimum bactericidal concentrations (MBCs) remaining below working concentrations for all disinfectants even in the presence of 20 per cent manure,^9^ implying effective concentrations could be achieved in a practical on-farm setting.

The work presented here uses prepared treponeme cultures inoculated on to hoof knife blades in two experiments. The objective of the first experiment was to test survival times of treponemes on hoof knife blades under aerobic conditions. The objective of the second experiment was to test a range of common disinfectants at working concentrations for removing viable treponemes from hoof knife blades.

## Materials and methods

### Treponeme culture preparation and inoculation on to hoof knives

Two strains of BDD-associated treponeme bacteria were used: T320A (*Treponema phagedenis*-like phylogroup) and T3552B (*T pedis*). Both were previously identified as associated with BDD lesions and were cultured as previously described.^4^ These were diluted to standardised concentrations (optical densities),^10^ and 0.5-ml cultures were applied to one side of each hoof knife blade (Aesculap VC300/VC305).

### Sampling

To act as positive controls in both studies, swabs (Copan Italia, Italy) were taken from blade surfaces two minutes after application of cultures and placed into liquid medium (oral treponeme enrichment broth with 10 per cent fetal calf serum).

For the survival study, samples were taken after a series of waiting times (10 minutes, then one hour, two hours, four hours and 18 hours). One swab was inoculated into liquid medium and transferred to an anaerobic cabinet as soon as possible; a second swab was stored at −20°C for direct detection of treponemes by nested PCR. For the disinfection study, swabs were taken in the same manner after blades were immersed for 20 seconds in one of the following disinfectants: 2 per cent (w/v) Virkon^®^ (DuPont, Wilmington, USA), 2 per cent (v/v) sodium hypochlorite, 2 per cent (v/v) glutaraldehyde, 5 per cent (w/v) copper sulphate or 1 per cent (v/v) FAM30^®^ (Evans Vanodine, Preston, UK). Since all these chemicals are diluted in water, the experiments included water only for comparison. For each disinfectant and strain, treponemes were inoculated in batches of five on to at least 15 different knives across a minimum of three different days.

### Phase contrast microscopy

All cultures were examined weekly for six weeks using phase contrast microscopy. Cultures were considered positive for treponeme growth if at least 10 treponemes with some motility were visible per field of view. Replicates that did not meet these criteria in the positive control culture by week six were discarded.

### DNA extraction

Genomic DNA was extracted from cotton swabs using a DNeasy Mini Kit (Qiagen, UK) according to the manufacturer’s instructions, and from cultures using Chelex resin (Bio-Rad, UK).^11^ All samples were stored at −20°C for testing by nested PCR.

### PCR assays

Nested PCR assays specific for each BDD treponeme phylogroup were carried out as previously described, with an initial step using universal 16S rRNA primers, followed by a phylogroup-specific nested PCR step, resulting in 300–500 bp products.^5^

## Results

### Treponeme survival on hoof knife blades

It was consistently possible to culture both strains of treponeme from hoof knives for up to two hours postinoculation (PI) (two of three replicates were positive using T320A, and three of three replicates were positive using T3552B). Treponeme growth was visible by phase contrast microscopy after one week, and in all cases nested PCR on genomic DNA extracted from these cultures confirmed microscopy findings. After four hours PI, treponemes could not be detected in culture, either by weekly phase contrast microscopy or by PCR testing of cultures after six weeks. All samples remained positive by direct PCR testing of swabs for the full 18 hours PI for treponeme strains (table 1).

**Table 1:** Survival time for two strains of treponeme inoculated on to hoof knife blades as determined by direct PCR, phase contrast microscopy and PCR of cultures after six weeks of incubation (three replicates)

Treponeme strain (phylogroup)	T320A (*Treponema phagedenis*-like)			T3552B (*Treponema pedis*)		
Sampling time postinoculation	PCR positive swabs	Phase contrast microscopy positive cultures	PCR positive cultures	PCR positive swabs	Phase contrast microscopy positive cultures	PCR positive cultures
10 minutes	3/3	3/3	3/3	3/3	3/3	3/3
1 hour	3/3	3/3	3/3	3/3	3/3	3/3
2 hours	3/3	2/3	2/3	3/3	3/3	3/3
4 hours	3/3	0/3	0/3	3/3	0/3	0/3
18 hours	3/3	0/3	0/3	3/3	0/3	0/3

### Disinfection of hoof knife blades

Three disinfectants completely prevented visible treponeme growth under laboratory conditions, as determined by phase contrast microscopy: 1 per cent FAM30^®^, 2 per cent Virkon^®^ and 2 per cent sodium hypochlorite. When using nested PCR of cultures after six weeks as an outcome, 1 per cent FAM30^®^ eliminated all detectable DNA, while there was detectable DNA in one of 13 T320A cultures postdisinfection with 2 per cent Virkon^®^ and two of 15 T320A cultures postdisinfection with 2 per cent sodium hypochlorite, suggesting some limited growth. Water was the least effective, leading to visible treponeme growth in 16 of 28 cases and positive PCRs from cultures in 19 of 28 cases.

Two per cent Virkon^®^ and 2 per cent sodium hypochlorite yielded the best DNA removal as determined by direct PCR of swabs in terms of removing/destroying all bacterial DNA in 18 of 26 and in 20 of 31 cases, respectively, while 1 per cent FAM30^®^ did not destroy bacterial DNA by this measure (table 2). Water resulted in positive results for treponemal DNA presence by direct PCR in 27 of 28 cases (table 2).

**Table 2:** Efficacy of disinfectants (20-second exposure time) against BDD treponemes on hoof knife blades determined by direct PCR, phase contrast microscopy and PCR of cultures after six weeks of incubation

Treponeme strain (phylogroup)	T320A (*Treponema phagedenis*-like)			T3552B (*Treponema pedis*)		
Disinfectant	Postdisinfection					
	PCR positive swabs	Phase contrast microscopy positive cultures	PCR positive cultures	PCR positive swabs	Phase contrast microscopy positive cultures	PCR positive cultures
Water	10/12 (83.3%)	6/12 (50.0%)	6/12 (50.0%)	16/16 (100%)	10/16 (62.5%)	13/16 (81.3%)
5% copper sulphate	13/16 (81.3%)	1/16 (6.3%)	4/16 (25.0%)	11/17 (64.7%)	1/17 (5.9%)	2/17 (11.8%)
1:100 FAM30	11/11 (100%)	0/11 (0.0%)	0/11 (0.0%)	12/12 (100%)	0/12 (0.0%)	0/12 (0.0%)
2% glutaraldehyde	15/15 (100%)	2/15 (13.3%)	6/15 (40%)	11/11 (100%)	0/11 (0.0%)	0/11 (0.0%)
2% sodium hypochlorite	10/15 (66.7%)	0/15 (0.0%)	2/15 (13.3%)	1/16 (6.3%)	0/16 (0.0%)	0/16 (0.0%)
2% Virkon	6/13 (46.2%)	0/13 (0.0%)	1/13 (7.7%)	2/13 (15.4%)	0/13 (0.0%)	0/13 (0.0%)

BDD, bovine digital dermatitis.

## Discussion

Here, viable BDD treponemes survived on hoof knife blades under aerobic laboratory conditions for two hours, which is probably key to their apparent ability to be transmitted between animals during foot-trimming.^7 8^ This work also demonstrates that three common disinfectants prevent visible growth of treponemes under anaerobic conditions in the laboratory: 1 per cent FAM30^®^, 2 per cent Virkon^®^ and 2 per cent sodium hypochlorite; 1 per cent FAM30^®^ also eliminated all detectable DNA from cultures. Interpretation of the direct PCR results from swabs is more challenging as positive results do not necessarily correspond to the presence of viable bacteria capable of growth in their normal biological context. Glutaraldehyde, for example, has fixative properties and could therefore be expected to preserve DNA while resulting in non-viable treponemes.^12^

Although the authors have demonstrated disinfectant effectiveness against a laboratory bacterial challenge, the present work does not mimic potential field contamination, and future field studies would be beneficial. It has been shown that BDD treponemes can be isolated in culture for three days from gloves contaminated during handling of sheep feet affected by contagious ovine digital dermatitis.^13^ Studies examining the role of gloves in BDD transmission may be beneficial in the future.

The finding of treponeme viability for two hours under aerobic conditions suggests it may be possible to transmit BDD treponemes on hoof knives both between cows in the same herd and among herds. It is therefore important to apply a disinfection protocol during foot-trimming, which should form part of a holistic approach to effective BDD control. The laboratory results presented here suggest that 1 per cent FAM30^®^, 2 per cent Virkon^®^ or 2 per cent sodium hypochlorite with 20-second contact time should be suitable for this purpose, although testing under field conditions would be beneficial.
